# Singapore’s 5 decades of dengue prevention and control—Implications for global dengue control

**DOI:** 10.1371/journal.pntd.0011400

**Published:** 2023-06-22

**Authors:** Soon Hoe Ho, Jue Tao Lim, Janet Ong, Hapuarachchige Chanditha Hapuarachchi, Shuzhen Sim, Lee Ching Ng

**Affiliations:** 1 Environmental Health Institute, National Environment Agency, Singapore, Singapore; 2 Lee Kong Chian School of Medicine, Nanyang Technological University Novena Campus, Singapore, Singapore; 3 School of Biological Sciences, Nanyang Technological University, Singapore, Singapore; Duke-NUS GMS, SINGAPORE

## Abstract

This paper summarises the lessons learnt in dengue epidemiology, risk factors, and prevention in Singapore over the last half a century, during which Singapore evolved from a city of 1.9 million people to a highly urban globalised city-state with a population of 5.6 million. Set in a tropical climate, urbanisation among green foliage has created ideal conditions for the proliferation of *Aedes aegypti* and *Aedes albopictus*, the mosquito vectors that transmit dengue. A vector control programme, largely for malaria, was initiated as early as 1921, but it was only in 1966 that the Vector Control Unit (VCU) was established to additionally tackle dengue haemorrhagic fever (DHF) that was first documented in the 1960s. Centred on source reduction and public education, and based on research into the bionomics and ecology of the vectors, the programme successfully reduced the *Aedes* House Index (HI) from 48% in 1966 to <5% in the 1970s. Further enhancement of the programme, including through legislation, suppressed the *Aedes* HI to around 1% from the 1990s. The current programme is characterised by 4 key features: **(i)** proactive inter-epidemic surveillance and control that is stepped up during outbreaks; **(ii)** risk-based prevention and intervention strategies based on advanced data analytics; **(iii)** coordinated inter-sectoral cooperation between the public, private, and people sectors; and **(iv)** evidence-based adoption of new tools and strategies. Dengue seroprevalence and force of infection (FOI) among residents have substantially and continuously declined over the 5 decades. This is consistent with the observation that dengue incidence has been delayed to adulthood, with severity highest among the elderly. Paradoxically, the number of reported dengue cases and outbreaks has increased since the 1990s with record-breaking epidemics. We propose that Singapore’s increased vulnerability to outbreaks is due to low levels of immunity in the population, constant introduction of new viral variants, expanding urban centres, and increasing human density. The growing magnitude of reported outbreaks could also be attributed to improved diagnostics and surveillance, which at least partially explains the discord between rising trend in cases and the continuous reduction in dengue seroprevalence. Changing global and local landscapes, including climate change, increasing urbanisation and global physical connectivity are expected to make dengue control even more challenging. The adoption of new vector surveillance and control tools, such as the Gravitrap and *Wolbachia* technology, is important to impede the growing threat of dengue and other *Aedes*-borne diseases.

## Author summary

A densely populated, highly urban tropical city-state with long-established populations of *Aedes aegypti* and *Ae*. *albopictus* mosquitoes, plus travel and trade links to all corners of the world, Singapore is ideally suited for dengue transmission. Singapore’s experience and rich surveillance data provide important insights for an increasingly large number of territories at risk of dengue epidemics. Decades of vector control efforts, focused on source reduction and surveillance, have successfully lowered the vector population, with accompanying reductions in the resident population’s seroprevalence to dengue. We propose that Singapore’s vulnerability to outbreaks is due to low levels of immunity in the population, constant introduction of viral variants, expanding urban centres, and increasing human density. The discord between the rising trend in reported cases and falling seroprevalence could be at least partly attributed to improved diagnostics and surveillance. Singapore’s evidence-based vector-control programme, which involves strong partnership between the public and private sectors, will continue to adapt to future challenges in this space.

## 1. Introduction

Dengue viruses (DENV) are hyperendemic in Singapore, with all 4 serotypes in co-circulation [1,2]. *Aedes aegypti*, the primary dengue vector, is well established in the tropical city-state, which has favourable conditions for breeding, including a year-round warm and humid climate and a highly urbanised population. In addition, the secondary vector, *Ae*. *albopictus*, is native to the country and thrives in the abundant foliage found throughout the island, including in built-up areas [3]. While *Ae*. *albopictus* is ubiquitous, *Ae*. *aegypti* is restricted to built-up areas. The geographical spread of dengue cases coincides with the spatial expansion of *Ae*. *aegypti*, and dengue cases were found to be associated with the percentage of *Aedes* breeding sites with presence of *Ae*. *aegypti* [3]. While *Ae*. *aegypti* was also the primary vector of a Zika outbreak in 2016 [4], *Ae*. *albopictus* played a key role in chikungunya transmission in Singapore in 2008 and 2013 [5,6]. Despite the 2 outbreaks, chikungunya seroprevalence remains low at 1% to 5% and showed a nonsignificant increase from 2009 to 2013 [5]. There has been no evidence of sustained transmission of chikungunya and Zika in Singapore since 2015 and 2018, respectively, though importations have been detected. However, given the presence of both vectors, Singapore remains vulnerable to chikungunya and Zika outbreaks.

**Fig 1:**
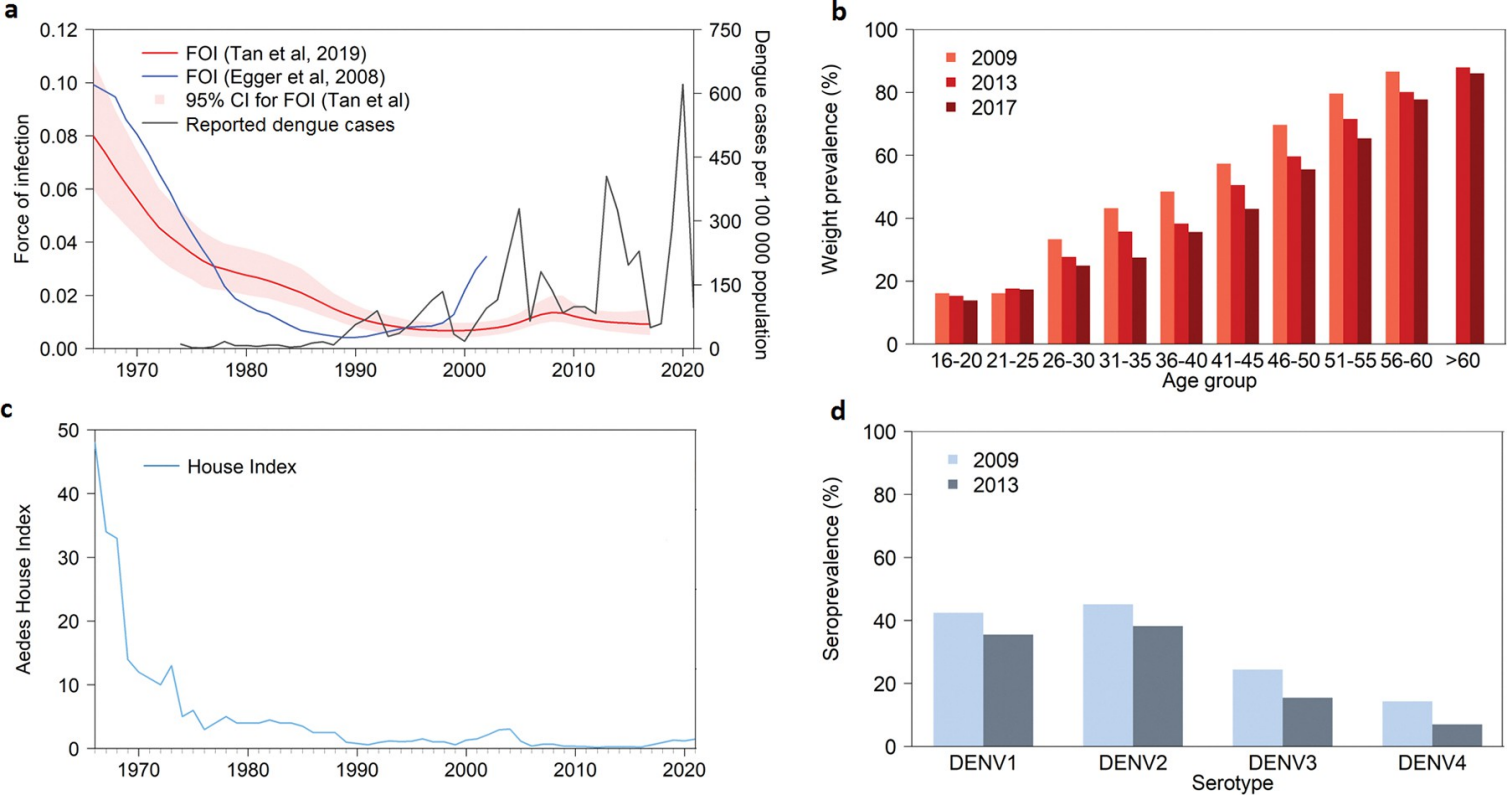
Epidemiologic and entomologic indicators of dengue transmission. (a) Dengue FOI from [13,14] with associated 95% credible intervals plotted where available, together with the number of reported dengue cases from the 1960s to 2021. (b) Seroprevalence of dengue across adolescent and adult age groups over 2009, 2013, and 2017 [13,15]. (c) *Aedes* House Index from the 1960s to 2021 ([11,12] and NEA internal data). (d) Prevalence of serotype-specific neutralisation antibodies among 16–60 year olds in 2009 and 2013 [15]. DENV, dengue virus; FOI, force of infection; NEA, National Environment Agency.

This paper aims to outline and explain the dengue epidemiological situation in Singapore from the 1960s to the present. While anti-malarial control efforts had been in place since 1921 with the enforcement of the Destruction of Mosquitoes Ordinance [7], vector control efforts were greatly stepped up when the Vector Control Unit (VCU) was formed in 1966 to address the growing number of dengue haemorrhagic fever (DHF) cases. The first 2 decades following the start of active vector source reduction efforts led to large decreases in the vector population and reported dengue cases, but subsequent decades saw high rates of reported cases (Fig 1A) despite a successful vector control programme coupled with decreasing population seroprevalence to dengue (Fig 1B). Evidence suggests that the paradoxical situation is caused by a reduced population immunity, diverse array of virus lineages and improvements in case notifications and diagnosis rates, among other possible factors. We subsequently discuss the evolution of the country’s dengue prevention efforts over the preceding 5 decades and highlight some of the lessons learned.

### 1.1 Increased dengue cases amid declining *Aedes* house index and force of infection

***Aedes* House index (HI) sustained at low level** While the earliest local epidemic of dengue fever (DF) was reported in 1901 [8] and sporadic epidemics were known to occur in Malaya and Singapore over the next 5 decades, it was only in the 1960s that the disease’s severity was recognised due to recurrent outbreaks of DHF [9,10]. The DHF outbreaks prompted the formation of the VCU in 1966 to tackle the high *Aedes* population in the country. From the outset, vector control centred on research to understand the bionomics and ecology of the vectors, which provided the basis for entomological surveillance, source reduction, and public education. At the same time, Singapore implemented infrastructural development and enhanced environmental management, with a public housing programme that improved the living conditions of the population, a holistic waste management programme, and regular campaigns to keep the city clean and mosquito-free. Together with the vector control programme, these efforts successfully reduced the *Aedes* HI from 48% in 1966 to <5% in the 1970s [11,12]. Further enhancement of the programme, including the passage of legislation that penalises homes found to have vector breeding habitats with immature larvae and pupae, has further suppressed the *Aedes* HI to around 1% since the 1990s (Fig 1C).

**Dengue seroprevalence and force of infection maintained at reduced levels** Concomitant with the decrease in *Aedes* HI, dengue seroprevalence among the resident population has also declined. The seroprevalence among youths between 15 and 19 years old plunged from 70% in the early 1980s to <20% since a survey in 1998 [15]. Current seroprevalence is significantly lower than the 39% to 92% found in the same age group in several endemic countries [16–19]. More recent local seroprevalence surveys conducted in 2009, 2013, and 2017 revealed continuing but more gradual decline in dengue seropositivity in all age groups, except among young adults between 21 and 25 years old (Fig 1B) [13]. The dengue force of infection (FOI) modelled using these data showed an approximate 10-fold decrease from >0.1 per year in the 1960s to about 0.01 since the 2010s (Fig 1A) [13,14]. This is consistent with the shifting of reported disease from predominantly paediatric cases in the 1960s to 1970s to young adults in recent decades [11,20,21]. Taken together, the evidence suggests that dengue incidence is substantially lower today compared to the 1960s, and not higher than the 1980s, contrasting with the trend observed among reported cases which has risen sharply since the 1990s with more frequent outbreaks (Fig 1A). The increase in reported dengue cases came against a backdrop of declining hospitalisation rate and continued low case fatality rate for dengue patients, which has been attributed to early diagnosis and improved management of patients by primary care physicians [22].

## 2. Potential factors contributing to the increase in reported dengue cases

Below, we delineate 6 potential factors that could have contributed to the increase in reported dengue cases against the backdrop of a suppressed vector population and reduced seroprevalence in Singapore.

**Low level of immunity among the population** Despite a suppressed *Ae*. *aegypti* population and an associated reduction in the force of infection (Fig 1A and 1C), Singapore remains vulnerable to outbreaks. A key driver is the low herd immunity of the population, evident from the continuous reduction in dengue seroprevalence across different age groups and the low prevalence of neutralising antibodies for each serotype among adults in Singapore (Fig 1B and 1D) [13,15]. Prevalence of antibodies against DENV1 and DENV2 are higher (at 35.8% and 36.4% for DENV1 and DEN2 among 16 to 60 year olds in 2013, compared to 15.4% and 7.7% for DENV3 and DENV4), consistent with these being the more common serotypes circulating in Singapore as determined by decades of virus surveillance [15,23]. Considering the high likelihood of cross-reactive antibodies across serotypes [24], actual immunity levels are likely to be lower than estimated. Paradoxically, the low human immunity to dengue caused by a suppressed vector population could counteract the reduced risk offered by the latter and could render the human population more sensitive to any increase in vector abundance and new virus strain introductions.

**Increased viral diversity due to Singapore’s increasing connectivity** As a highly globalised trade- and tourism-dependent country, a high number of DENV variant introductions is to be expected. Consequentially, Singapore’s highly diverse DENV population has been maintained over the years with fluctuations in overall composition [2,12,25]. The number of international visitor arrivals to Singapore grew over 10-fold from <100,000 in 1964 to >15 million in 2013 [26]. Prior to border restrictions due to the Coronavirus Disease 2019 (COVID-19) pandemic, the number of local residents (citizens and permanent residents) who travelled overseas rose from 520,000 (10% of population) in January 2011 to 760,000 (13% of population) in the same month in 2020 [27]. Border restrictions in 2020 and 2021 led to a decline in dengue viral diversity, as evidenced by the absence of DENV1 among serotyped samples from November 2020 to June 2022, a previously common serotype in constant circulation within Singapore over the preceding 3 decades. As intense serotyping was performed for an average of 30% of all reported cases each week during that period, the absence of DENV1 for 7 months suggests extinction of the serotype during border closure, after which DENV1 reemerged.

Multiple introductions offer ample opportunities for the selection of viruses with high epidemic potential. A DENV2 cosmopolitan clade replacement event in 2007 showed that small genetic shifts of 9 amino acid substitutions were associated with a local outbreak [1,28]. Infection studies revealed higher replication rates of the new clade in vector and mammalian cell lines [1,28], resulting in shortened extrinsic incubation period (EIP) in mosquitoes and possibly increase in viraemia levels in patients, respectively. Other examples of dengue epidemics following the introduction of novel viral genotypes include the 2009 epidemic in Sri Lanka [29] and the 2014 to 2015 epidemics in Taiwan [30].

**Improved case ascertainment rate through diagnostic improvements** While low population immunity and high viral diversity shed light on the vulnerability of Singapore to outbreaks, they do not explain the discord between a continuing reduction in seroprevalence and increasing dengue incidence rate. We propose that better case ascertainment and notification through improved surveillance and diagnostics likely contributed to the increase in reported incidence rates in the last 5 decades (Table 1). While DHF was made legally notifiable in 1972, DF was in 1977 [20]. Though the case ascertainment rate was likely improved by law, it remained very limited as diagnosis was based on clinical assessment and most dengue cases had undifferentiated symptoms [31]. Dengue serology testing was made available globally in the 1980s [32] but usage in Singapore was limited to hospitals among the more severe cases. Even as late as 2006, 70% of dengue cases were reported by hospitals with the rest by primary healthcare [22]. Undifferentiated fever cases caused by dengue seen by primary healthcare could thus be underreported. To improve reporting, a programme was launched to make PCR-based early diagnostic tests available at a hospital in 2003 and at the Environmental Health Institute (EHI) in 2005 for private primary healthcare clinics [1,33]. However, capacity was only limited to a few laboratories. In 2008, a campaign encouraging clinical laboratories to use commercial dengue nonstructural protein 1 (NS1) rapid tests was launched and these tests have since become widely used by all clinical laboratories in Singapore [34]. These advances in dengue diagnostics and improvement in the healthcare system coincided with the progressive increase in reported dengue cases in Singapore, during which the FOI remained low at around 0.01. The estimated case-ascertainment rate rose from 1 in 14 infections from 2005 to 2009 to 1 in 6 infections in 2014 to 2017 [13].

**Table 1:** Key events related to dengue prevention efforts in Singapore.

Year	Key events
**1960s**	
1960s	Ongoing public housing programme to clear slums and provide affordable high-rise apartments to residents
1966	Formation of VCU under the MOH Launch of pilot study to control *Aedes* population in Geylang area
1967	Launch of study on *Aedes* ecology and bionomics
1968	Passage of DDBIA
1969	Launch of Keep Singapore Clean and Mosquito Free campaign First use of a modified ovitrap for *Aedes* control at Paya Lebar airport
**1970s**	
1972	Formation of the Ministry of the Environment (ENV) Transfer of Vector Control Unit (VCU) from MOH to ENV DHF made a notifiable disease
1973	Large outbreak of DF/DHF with 1,187 reported cases First use of chemical pesticides to control *Aedes* in Singapore
1977	Passage of IDA DF made a notifiable disease
**1980s**	
1980s	Introduction of dengue-specific IgM and IgG serology tests for diagnosis
**1990s**	
1997	Annual reported dengue incidence surpasses 100 per 100,000 for the first time
1998	Passage of CVPA which empowers public health officers to conduct source reductions
**2000s**	
2002	Formation of NEA under ENV Consolidation of dengue prevention efforts under NEA
2005	Introduction of dengue Polymerase Chain Reaction test for diagnosis
2008	Nationwide introduction of dengue nonstructural protein 1 (NS1) ELISA for diagnosis
**2010s**	
2013	Introduction of the Gravitrap for *Aedes* sentinel surveillance
2016	Start of phased testing approach of releasing *Wolbachia*-infected male *Ae*. *aegypti* mosquitoes to control dengue at Tampines, Yishun, and Braddell Heights
**2020s**	
2020	Largest recorded dengue outbreak with 31,315 reported cases coincided with COVID-19 control measures (“circuit breaker”)

Main sources of information: [11,20,36–39].

COVID-19, Coronavirus Disease 2019; CVPA, Control of Vectors and Pesticides Act; DDBIA, Destruction of Disease Bearing Insects Act; DF, dengue fever; DHF, dengue haemorrhagic fever; ELISA, enzyme-linked immunosorbent assay; IDA, Infectious Diseases Act; MOH, Ministry of Health; NEA, National Environment Agency; VCU, Vector Control Unit.

This paradoxical trend highlights the limitations of a passive but changing case surveillance system to measure the long-term impact of dengue control programmes, a challenge shared by endemic countries globally. Case ascertainment helps to preempt and detect outbreaks and enables risk assessment and risk stratification for optimisation of dengue prevention efforts [35], thereby making surveillance a fundamental component of a vector control programme. However, using reported case counts to measure dengue burdens will be confounded by changing ascertainment rates, making periodic seroprevalence studies necessary for long-term impact assessment and triangulating the change in true transmission intensity over time.

**Persistent presence of *Aedes* and climatic and spatial factors** Singapore’s warm and humid climate year-round allows for favourable breeding and survival conditions for the *Ae*. *aegypti* vector. An increase in the ambient temperature between 25°C and 35°C accelerates the life cycle of mosquito vectors [40] and reduces the EIP of the dengue virus in the vector [41], thereby increasing the transmission potential of dengue virus [42]. For example, in Cairns, Australia, unseasonably warm temperatures above 30°C in late 2008 are believed to have shortened the EIP of DENV3 in *Ae*. *aegypti* and contributed to a 2008 to 2009 epidemic [43]. However, the relationship between mosquito survival and temperature is inverted U-shaped with optimum temperatures for *Ae*. *aegypti* and *Ae*. *albopictus* around 27.5°C and 21.5°C, respectively [44]. Extreme heat could thus be detrimental to dengue transmission through its inhibitory effect on vector survival [44]. This is supported by an analysis of recent dengue cases in Singapore that estimated that daily maximum temperatures above 31°C and heat waves (defined as weeks with 2 or more days exceeding the 90th percentile of maximum temperature) were associated with reduced dengue [45]. Singapore has warmed over the past decades, with the number of months per year with mean temperature above 27.5°C (the optimal temperature for the survival of *Ae*. *aegypti*) exhibiting a positive trend between 1980 and 2021 (as measured at Changi meteorological station) [46]. With warming facilitated by climate change and urban heat island effect due to high urbanisation with large contiguous built-up areas, dengue transmission may be exacerbated [47]. However, given that extremely high temperature is detrimental to vector survival, if temperatures continue to rise, Singapore might experience a reversal of dengue seasonality where less transmission occurs in the middle of the year when the temperatures are too hot and heightened transmission at year’s end with lower, more conducive temperatures.

**Fig 2:**
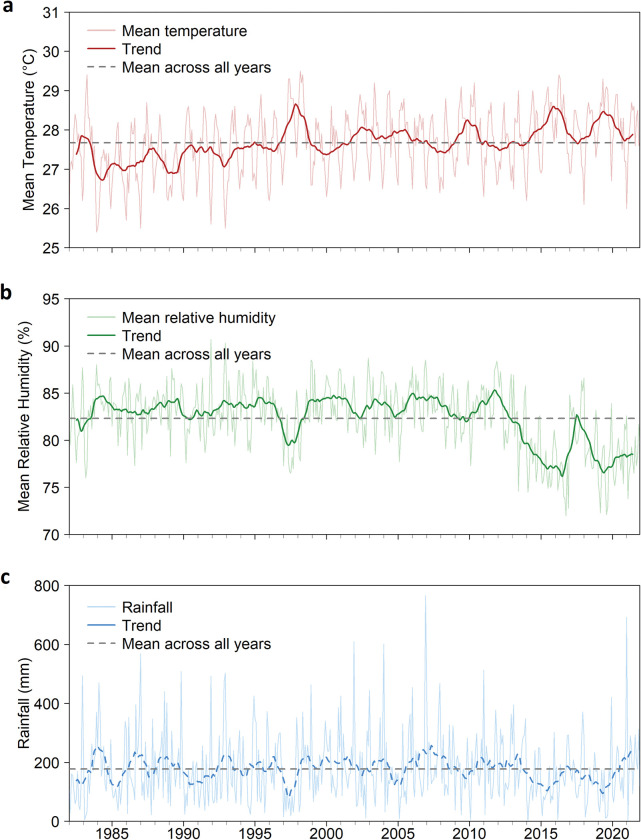
Recorded weather factors across time 1980 to 2022 including the mean, trend, and raw measurements for (a) temperature, (b) relative humidity, (c) rainfall at Changi Climate Station [46].

Relative humidity and rainfall are also associated with mosquito vector survival [44,48]. While rain is typically a risk factor for dengue [48], higher rainfall during the north-east monsoon could reduce dengue risk by flushing away outdoor habitats of the vectors [49]. In Singapore (Changi), precipitation from 1980 to 2021 remained nearly constant (Fig 2C) while the mean relative humidity had exhibited a downward trend since the 2010s (Fig 2B) [46], which might slightly attenuate the risk of dengue transmission at that location.

**Increasing urbanisation and population density increases human–vector interactions** Human population density in Singapore has increased from about 3,200 persons per km^2^ in 1965 to 7,600 persons per km^2^ in 2022 [50], with the population density reaching close to 50,000 people per km^2^ in some areas [51]. With a highly dense population living in built-up areas and expansion of such areas, there are increasingly more breeding and biting opportunities for the anthropophilic *Ae*. *aegypti*, which breeds in man-made receptacles [52]. The risk is exacerbated by the aging of buildings and numerous construction activities, both of which have been shown to be risk factors for dengue transmission and/or *Ae*. *aegypti* abundance [53,54].

**Novel work arrangements associated with dengue epidemiological changes in 2020** In 2020, Singapore experienced an unprecedented approximately 35,000 case dengue outbreak, coinciding with the first year of the COVID-19 pandemic. A study showed that non-pharmaceutical interventions to reduce SARS-CoV-2 transmission, in particular, the partial lockdown from April to June 2020, was associated with an excess of dengue cases among working adults [55]. The study postulated that the outbreak was exacerbated by the working population spending prolonged periods of time in naturally ventilated homes rather than in air-conditioned offices or workplaces; this would have increased the chances of interaction with anthropophilic *Ae*. *aegypti*, which tends to breed and dwell in and around homes [55,56]. This shows the risk of home-based infection and contradicts an older hypothesis that dengue transmission in Singapore was more likely to occur outside homes [20]. In contrast, the same measures led to a decrease in dengue transmission among migrant workers who are largely employed in the local construction industry [56]. Confinement to dormitories during the lockdown, instead of working in construction sites, lowers the risk of dengue among workers—an observation that is consistent with evidence that showed higher dengue risk in construction sites [53].

Similar to the migrant worker population in Singapore, COVID-related lockdowns were associated with attenuated dengue transmission in Southeast Asia and Latin America [57]. This highlights differences in relative transmission risk between work and home locations across dengue endemic countries and populations and illustrates changing epidemiology through time and through work arrangements.

## 3. Evolution of Singapore’s dengue prevention efforts

To tackle the persistent challenge of dengue, Singapore has refined and strengthened its dengue prevention strategy since 1965 through the 2000s.

From its inception, Singapore’s dengue prevention programme has focused on environmental management and public education. In 1966, the first documented local pilot study in a township where a majority of the population lived in slums, thatched or zinc roofed houses, shophouses, or newly built public high-rise residential apartments showed the effectiveness of integrated source reduction and health education at reducing the *Aedes* population [36,58] (Table 1). The pilot project successfully reduced the *Aedes* HI from a mean of 16% to 2% [36], leading to wider, national adoption. The subsequent passage of the Destruction of Disease Bearing Insects Act (DDBIA) in 1968 strengthened legal powers of vector control personnel to inspect premises for potential vector breeding [36]. The Keep Singapore Clean and Mosquito Free campaign was launched in 1969 to further mobilise public support for vector control efforts [36]. The transfer of the VCU from the jurisdiction of the Ministry of Health (MOH) to the newly formed Ministry of the Environment (ENV) in 1972 reflected the recognition of a need to align vector control with environmental management in dengue prevention [20,38].

The critical features driving Singapore’s more than 5 decades of dengue control efforts—source reduction, vector surveillance, community education, and legislation—were established within the first 5 years of the programme. The evidence-based approach adopted in the 1960s has continued till today where policies and operations are guided by scientific studies and data [59,60]. Through the years, the system development has been guided by 4 key principles that are consistent with those recommended by the World Health Organization [61]: **(i)** inter-epidemic surveillance and control; **(ii)** risk-based prevention and intervention; **(iii)** coordinated inter-sectoral cooperation; and **(iv)** development and adoption of science and technology.

**Inter-epidemic surveillance and control enhanced by legislation** Vector, virus, case, and environmental surveillance and control are integrated to mitigate dengue transmission in Singapore. The approach is proactive and preemptive rather than reactive as efforts continue regardless of whether the country is experiencing a dengue epidemic or not, although the frequency of inspection increases during epidemics. While the sustained surveillance provide data for outbreak alerts, risk stratification and prioritisation of resources, the sustained control between epidemics aims to moderate their intensity by reducing vector population and baseline of cases which would serve as a springboard for dengue transmission when dengue season approaches.

Case and virus surveillance by the healthcare system is supported by the Infectious Diseases Act (IDA) that mandates the reporting of all dengue cases by clinicians and diagnostics laboratories. Since 2005, greater advocacy and the setting up of a network of primary healthcare providers have enabled earlier case detection and higher ascertainment rates. Besides halving the average vector control response time (time taken from fever onset to initiation of vector control) from 7 days in 2004 to 3.5 days in 2010, the estimated ascertainment rate increased from 1 in 14 infections in 2005 to 2009 to 1 in 6 infections in 2014 to 2017 [13]. Circulating DENV populations are also monitored through a virus surveillance programme [1], where a subset of blood samples from suspected and test-positive dengue patients are subjected to serotype and genotype analyses on a weekly basis. This provides timely updates on the composition and distribution of DENV to facilitate resource allocation for dengue control operations and enables preemptive alerts in case of an outbreak signal such as a switch in the predominant serotype [1] and novel strains taking hold in the population which are associated with outbreaks in Singapore [12].

Vector surveillance and control is supported by the Control of Vectors and Pesticides Act (CVPA) [20,62] that legally empowers public health officers to conduct routine house inspections. Inspections and source reduction exercises form the bedrock of vector-based interventions in Singapore (Table 1), and in recent years can total >1,000,000 annually [63]. These involve checking premises and their surrounding areas for receptacles that can collect water and breed mosquitoes. Checks are conducted year-round and intensify before the traditional dengue season approaches to detect and remove breeding, create community awareness, and identify potential breeding sites due to infrastructural defects for rectification [11,38]. Particular attention is paid to reducing the *Aedes* population and the number of dengue cases before the traditional dengue season between May and October [11,38]. Households averaging 3 inspections per annum were associated with reduced odds (adjusted odds ratio: 0.49 [95% CI: 0.38 to 0.63]) of mosquito larval habitat reports [63]. Together with the penalty imposed on premise owners found to harbour vector breeding, the inspection system motivates premises owners and occupiers to be more vigilant against mosquitoes breeding [63].

More recently, Gravitraps, developed by the EHI, a public health laboratory within the National Environment Agency (NEA) [64], have been mass-deployed for *Aedes* surveillance. The more than 70,000 trap network distributed island-wide covering about 80% of residences provides a 3D spatial picture of mosquito populations [65] that serves several objectives. The Gravitrap *Aedes aegypti* Index (GAI), based on the number of adult female *Ae*. *aegypti* caught per Gravitrap deployed in a locality, is a proxy for vector abundance and was found to be associated with dengue risk [59]. The index thus provides useful information on potential dengue risk to guide vector control operations [65], especially in inter-epidemic periods where reported dengue case counts are low and reduction in mosquito populations becomes the primary objective. Besides guiding vector control, the GAI is used to alert residents around areas with high vector abundance through the deployment of banners and visualisation of data on official applications and webpages. The GAI system also allows evaluation of control tools, such as the *Wolbachia* technology under pilot deployment in Singapore. By luring and removing gravid female *Ae*. *aegypti* mosquitoes, Gravitraps also play a role in reducing the *Aedes* population and were associated with an estimated 30% dengue risk reduction in the area of deployment [59].

**Risk-based prevention and intervention through modelling and data analytics** The adoption of data analytics tools allows for risk-based vector-control resource allocation. NEA has an integrated dengue alert surveillance system that combines information from clinical and laboratory diagnoses, circulating viral genotypes, *Aedes* population, and ecological parameters [66]. Dengue forecast models using machine learning and statistical methods that provide early warning of outbreaks to guide policymakers and NEA’s vector control operations up to 3 months in advance [60,67]. In case of an outbreak signal, stakeholders are alerted and national dengue campaign brought forward to collectively prepare for outbreaks and preemptively reduce *Aedes* population on the island. Annually, a spatial risk model is also built using information such as historical dengue burden, age of buildings, and amount of vegetation in an area to stratify transmission risk and guide resource allocation [68]. NEA has also adopted novel indices for dengue surveillance to better inform public health operations and channel resources to high-risk areas, such as estimating the effective reproduction number for dengue, which informs disease transmissibility in real time [42].

**Coordinated inter-sectoral cooperation and adaptive communication strategies to reduce potential *Ae*. *aegypti* breeding** With the dynamic urban landscape providing an array of breeding opportunities for *Ae*. *aegypti*, coupled with limited resources for vector control, it is critical to engage multiple stakeholders on good housekeeping and essential vector control measures to be conducted at their premises. Besides ensuring that the activities of stakeholders do not compromise source reduction and vector control efforts, this also encourages a ground-up, concerted, and proactive approach to protect more individuals against dengue.

Apart from the MOH, NEA works closely with other ministries and government agencies, academia, and the public to advance dengue prevention and control efforts [38]. First, the initiation of the Inter-Agency Dengue Task Force, which comprises Town Councils and key stakeholders from various government agencies, allows for regular situational dengue updates and sharing of vector control practices [38]. Also, as construction sites were estimated to have significantly higher risk of dengue transmission [53], NEA works closely with the Singapore Contractors Association to assist contractors in minimising mosquito breeding [38] and mandates the employment of environmental control officers to prevent environmental problems such as stagnant water for vector breeding [69]. A final example of inter-sectoral collaboration is NEA’s work with the Housing and Development Board (HDB) and the Building and Construction Authority (BCA) to re-design high-rise apartment blocks without roof gutters and replace cylindrical bamboo pole holders in older flats (where clothes are hung out to dry) with brackets, which helped to eliminate 2 key locations where rainwater tended to collect and allow vector breeding [38,62].

Public engagement to mobilise communities against mosquito breeding has also adapted to the changing population demographics. The original Keep Singapore Clean and Mosquito Free campaign of 1969 involved the distribution of leaflets and organisation of group competitions to educate the public about the sources of mosquito breeding [70]. Since then, public engagement has intensified through annual National Dengue Prevention Campaigns, with launches timed to precede the forecasted dengue peak each year [38]. Informational banners are colour-coded with traffic light signals to inform residents about the dengue risk level in their locality and posters incorporate graphic elements designed to attract public attention. Messages are short and sometimes accompanied by mnemonics to increase effectiveness [71,72].

To complement traditional media, social media is also leveraged to expand the reach of dengue prevention messages and target different audiences. NEA has accounts with Facebook, Instagram, Twitter, and TikTok, and uses these platforms to rapidly disseminate the latest information about the dengue situation. They also allow the tracking of user engagement of those posts, enabling fine-tuning of messages to suit targeted audiences. In 2011, NEA launched the myEnv mobile application that alerts users of dengue clusters and high *Ae*. *aegypti* populations so that the public can take the necessary precautions [73]. A newer version of the app was launched in 2021. These adaptations in public engagement have helped to increase communication efficiency and maximise the reach and impact of dengue prevention messages.

**Adoption of science and technology to improve dengue control** Given the already low *Ae*. *aegypti* population, attempts to achieve further reductions with conventional tools will yield diminishing returns, particularly against the backdrop of an increasingly conducive environment. Cost-effective advancement in dengue control thus requires a paradigm shift in strategy.

NEA has since 2016 been piloting the *Wolbachia*-based incompatible insect technique (IIT) [74,75], involving releases of *Wolbachia-*infected male *Ae*. *aegypti* mosquitoes to suppress urban vector populations. The potential of the technology, which has also garnered strong support from the community [76,77], was demonstrated by a 98% suppression of *Ae*. *aegypti* populations and 88% reduction of dengue incidences in pilot sites [75]. Automation solutions have been developed to ramp up rearing and releases of *Wolbachia* males; releases currently cover 50 km^2^ of residential areas encompassing around 1 million residents, almost 20% of Singapore’s population [78]. A hypothetical national IIT programme was estimated to be cost-effective, at about $50,000 to $100,000 per disability-adjusted life year (DALY) averted in 2010 USD [79], and further automation enhancements are ongoing to improve cost-effectiveness. A randomised controlled trial is also underway to provide statistically robust data on the epidemiological impact of *Wolbachia*-based IIT in Singapore [80].

*Wolbachia*-based IIT, instead of the introgression approach (which seeks to gradually replace the wild-type *Ae*. *aegypti* population with a *Wolbachia* infected one and involves the release of male and female *Wolbachia* infected *Ae*. *aegypti* into the field), has been adopted as it harmonises with the focus on vector suppression in the country and has greater social acceptance as biting females are not released. The introgression approach may not be effective in Singapore as an uncontrolled increase in *Wolbachia* infected *Ae*. *aegypti* population will negate the partially reduced vector competence [81]. As *Ae*. *aegypti* population in Singapore is very low, such increase is very likely if the community slackens source reduction efforts. Viral evolution to resist *Wolbachia*-mediated blocking in female *Ae*. *aegypti* is also likely in the long term [82].

The successful translation of research and development into dengue control operations is facilitated by a dedicated research programme that is closely integrated with the control programme. Besides *Wolbachia*-based IIT, other examples include the Gravitrap Surveillance system, risk-based inspections, and the use of drones for inspection for mosquito breeding habitats [83–85].

## 4. Implications for the globe: Singapore’s dengue epidemiology illustrates the challenge of dengue control

Complex dengue viral dynamics and diversity are not unique to Singapore. Global connectivity has facilitated continuous introduction and exchange of dengue viruses across borders, with dengue transmission taking hold where the efficient vector *Ae*. *aegypti* thrives and environmental conditions are favourable [86–90]. Such introductions, coupled with in situ evolution, play key roles in shaping the local dengue epidemiological landscape and provide ample opportunities for the selection of viruses with high epidemic potential [2,25]. While the mosquito vector remains dengue’s primary driver of spread across small spatial scales, viral dynamics suggest that the high mobility of infected human hosts play a key role in driving outbreaks across countries and regions [86,88,91].

In the absence of an effective vaccine or antiviral, vector control remains the only means of moderating the impact of the disease. However, an epidemiologically effective vector control programme suppresses human population immunity towards the virus, which in turn demands further suppression of the vector population to arrive at a new equilibrium to prevent an outbreak. This feedback-looped relationship between vector population and human immunity highlights the paradoxical challenge of dengue control, which is expected to play out in any locale with decades of successful vector-based dengue control, including those using *Wolbachia* for vector population suppression or introgression [75,92]. This is consistent with a modelling study suggesting that reductions in dengue incidence, effected by successful vector control or modification, including *Wolbachia* introgression approach, would gradually be eroded unless the intervention is implemented at higher intensity [93,94].

Another implication is that successful vector control and low FOI tends to demographically shift dengue from a paediatric disease to one of young adults, thus reducing mortality and morbidity associated with infection of vulnerable paediatric populations. However, a further reduction of the FOI could shift dengue to primarily affect older adults, in whom preexisting medical conditions are common and may increase the risk of severe dengue, as experienced in Singapore [20].

Singapore’s experience highlights the global need for novel vector control tools that are more effective than classical approaches, as well as nonvector-based intervention tools such as tetravalent vaccines that raise population immunity, and antivirals which reduce the rate of transmission from infected hosts to vectors. Although the Dengvaxia vaccine has been approved by a number of regulatory authorities, its adoption is limited due to its potential to predispose immunologically naïve recipients to severe dengue [95]. Because of the lack of safety data among older adults, its utility is further limited by the contraindication for individuals >45 years old [95,96]—a group that would greatly benefit from protection due to higher prevalence of comorbidities and which, ironically, would be suitable for vaccination as they are more likely to be seropositive for dengue. In the pipeline are new vaccine candidates and antiviral candidates [97–99], the success of which is urgently needed to reduce the burden of dengue for all age groups, including paediatric and elderly populations.

The conflicting trends between reported case numbers and FOI in Singapore highlight the limits of passive surveillance, especially in programmes that are progressively enhanced. Progress in vector control often occurs hand-in-hand with improvements in dengue surveillance, and the impact of vector control could be masked by improvements in case ascertainment. It is, therefore, important that evaluation and monitoring of programmes, both at local and global levels, include more accurate measures such as seroprevalence.

Singapore’s experience and rich data provide important insights for an increasingly large number of territories at risk of dengue epidemics. We have witnessed the geographical expansion of locations favourable for *Ae*. *aegypti* populations driven by the growth and expansion of cities [3] and climate change [100]. Warmer weather and longer summers, coupled with the proliferation of breeding habitats among artificial containers and urban infrastructure, will continue to create more opportunities for *Ae*. *aegypti* to expand its range. This is further exacerbated by increasing human population densities and the constant inflow of dengue-susceptible individuals into cities as a result of global migrations from non-endemic areas to endemic cities [52,101]. These challenges underscore the importance of considering mosquito breeding prevention in urban planning and building codes [102]. Such environmental protection measures, together with community collaboration, will be essential to complement any vector control tool, classical or novel, in the control of dengue and other *Ae*. *aegypti*-borne viruses [52,103]. A concerted effort by all endemic territories to control dengue is required to reduce opportunities for viral mutation and exchanges and consequently large epidemics.

## 5. Conclusions

Singapore has since the 1960s put in place an effective dengue control and prevention programme, leading to a consistent decrease in dengue seroprevalence and FOI till the present day. Paradoxically, the resulting low herd immunity, in addition to other factors such as introduction of new serotypes/clades, increased urbanisation and globalisation, has today contributed to exacerbated dengue risk. Improvements in surveillance and diagnostics have also resulted in an uptick in reported dengue cases despite the maintenance of low *Aedes* HI. Through public-private-people partnerships and leveraging the latest scientific and technological expertise in vector control, Singapore’s dengue vector control programme aims to continue to adapt to future challenges. Singapore’s experience and data could provide valuable insights on dengue epidemiology and control for the global community.
